# Optimized screening of DNA methylation sites combined with gene expression analysis to identify diagnostic markers of colorectal cancer

**DOI:** 10.1186/s12885-023-10922-2

**Published:** 2023-07-03

**Authors:** Zhen Ye, Guangle Song, Jianwei Liang, Shuying Yi, Yuqi Gao, Hanming Jiang

**Affiliations:** 1 Department of Health Management, The First Affiliated Hospital, Shandong Provincial Qianfoshan Hospital, Shandong First Medical University, Jinan, 250013 Shandong China; 2 Department of General Surgery, Tai’an City Center Hospital, Taian, 271000 Shandong China

**Keywords:** Hypermethylation, NaiveBayes, 10-fold cross-validation, Immune estimations, Colorectal cancer

## Abstract

**Background:**

The prognosis of patients with colorectal cancer is related to early detection. However, commonly used screening markers lack sensitivity and specificity. In this study, we identified diagnostic methylation sites for colorectal cancer.

**Methods:**

After screening the colorectal cancer methylation dataset, diagnostic sites were identified via survival analysis, difference analysis, and ridge regression dimensionality reduction. The correlation between the selected methylation sites and the estimation of immune cell infiltration was analyzed. The accuracy of the diagnosis was verified using different datasets and the 10-fold crossover method.

**Results:**

According to Gene Ontology, the main enrichment pathways of genes with hypermethylation sites are axon development, axonogenesis, and pattern specification processes. However, the Kyoto Encyclopedia of Genes and Genomes (KEGG) suggests the following main enrichment pathways: neuroactive ligand–receptor interaction, calcium signaling, and cAMP signaling. In The Cancer Genome Atlas (TCGA) and GSE131013 datasets, the area under the curve of cg07628404 was > 0.95. For the NaiveBayes machine model of cg02604524, cg07628404, and cg27364741, the accuracies of 10-fold cross-validation in the GSE131013 and TCGA datasets were 95% and 99.4%, respectively. The survival prognosis of the hypomethylated group (cg02604524, cg07628404, and cg27364741) was better than that of the hypermethylated group. The mutation risk did not differ between the hypermethylated and hypomethylated groups. The correlation coefficient between the three loci and CD4 central memory T cells, hematological stem cells, and other immune cells was not high (p < 0.05).

**Conclusion:**

In cases of colorectal cancer, the main enrichment pathway of genes with hypermethylated sites was axon and nerve development. In the biopsy tissues, the hypermethylation sites were diagnostic for colorectal cancer, and the NaiveBayes machine model of the three loci showed good diagnostic performance. Site (cg02604524, cg07628404, and cg27364741) hypermethylation predicts poor survival for colorectal cancer. Three methylation sites were weakly correlated with individual immune cell infiltration. Hypermethylation sites may be a useful repository for diagnosing colorectal cancer.

**Supplementary Information:**

The online version contains supplementary material available at 10.1186/s12885-023-10922-2.

## Introduction

Colorectal cancer (CRC) is the fourth deadliest cancer worldwide. In 2017, there were 1.8 million cases of colon and rectal cancer and 896,000 deaths worldwide [1]. Factors such as an aging population, obesity, lack of physical exercise, and smoking increase the risk of CRC, which accounted for 9.2% of all cancer deaths in 2018 [2]. In 2022, the estimated number of new cases of cancer in China exceeded 590 000, ranking second among all types of cancers, whereas that in the United States exceeded 160 000 [3]. Colorectal cancer imposes a heavy burden on the society.

Similar to other malignant tumors, the prognosis of patients with CRC is related to early detection, which is still the most effective clinical strategy for disease recovery when combined with accurate diagnosis and staging. A commonly used biomarker for CRC screening is the carcinoembryonic antigen (CEA). However, the sensitivity and specificity of CEA are poor. In 50 patients with CRC, the sensitivity of CEA detection was only 70% [2]. In another study, recurrent CRC was detected, with the most common CEA threshold of 5 µg/L. The sensitivity was 71%, and the specificity was 88% [4]. In a study using 1027 samples, the sensitivity of CEA in detecting CRC was 95%, and the specificity was only 43.9% [5]. In the absence accurate biomarkers, CEA monitoring of CRC should be combined with clinical, endoscopic, and imaging monitoring to improve accuracy [6].

Novel diagnostic methods for CRC have recently been developed. In one study, the sensitivity and specificity of tetraspanin 1 in the diagnosis of colon cancer were 75.7% and 66.7%, respectively [6]. In another study, circulating Pir-28,876 exhibited 75% sensitivity and 70% specificity for the diagnosis of colon cancer [7]. A fecal immunochemical test had a sensitivity of less than 40% and a specificity of more than 90% for the diagnosis of CRC [8]. In an additional study, the sensitivity and specificity of circulating cell-free DNA in the diagnosis of multiple tumors, including CRC, were 67.3% and 99.3%, respectively [9]. Furthermore, peripheral blood and immune cell markers could classify colon cancer vs. healthy populations with a sensitivity of 91% and specificity of 88% [10]. A novel, non-invasive CRC screening tool based on bacterial fecal biomarkers has also been developed, which, when combined with fecal immunochemical tests, can reduce false positive rates, with sensitivity and specificity estimates of 83% and 80%, respectively [11]. A meta-analysis revealed that the pooled sensitivities of fecal immunochemical tests were 73% for stage I CRC detection and 80%, 82%, and 79% for the detection of CRC stages II, III, and IV, respectively [12]. Biomarker screening of fecal microbiota can also be used to detect colon cancer [13]. Through verification using independent samples, the combination of microbial ratios of *Fusobacterium nucleatum/Bifidobacterium* (Fn/Bb) and *Fusobacterium nucleatum*/*Faecalibacterium prausnitzii* (Fn/Fp) was found to detect colon cancer with a specificity of 90.2% and a sensitivity of 90% [14]. Finally, another study established a model based on four differentially expressed microRNAs (Mir-28-3p, LET-7E-5p, Mir-106a-5p, and Mir-542-5p), which was validated using The Cancer Genome Atlas (TCGA) colorectal tissue dataset, with 99.7% sensitivity and 90.9% specificity [15].

Changes in the methylation state of genes, including a decrease in the overall methylation level of the genome and an abnormal increase in the local methylation level of CpG islands, which lead to genomic instability, are important in tumor development [16]. The single serum DNA methylation marker CG10673833 was found to exhibit a high sensitivity of 89.7% and specificity of 86.8% for the detection of CRC and precancerous lesions in 1493 high-risk individuals [17]. Furthermore, a DNA methylation panel could accurately distinguish colon cancer subtypes with more than 90% accuracy [18]. With progress in research, DNA methylation has great potential to serve as a disease biomarker in the future [19–21]. DNA methylation has been reported as a tumor biomarker in a large number of published studies; however, only 14 DNA methylation markers have been translated into commercial applications [22]. Furthermore, only two tests for CRC screening have been approved by the U.S. Food and Drug Administration, one of which involves the testing of stool samples (Cologuard; NDRG4 and BMP3), and the other involves the testing of serum [22]. Determining the exact location of CpG islands of clinically related genes is an important step in the development of DNA methylation biomarkers. Currently, DNA methylation markers are successfully applied in clinical practice in less than 1% of cases.

Some DNA methylation sites have a poor cancer prognosis and are even related to cancer progression. Selected DNA methylation sites can be used as potential targets for cancer therapy. Furthermore, DNA methylation inhibitors have become the main drugs used for the treatment of some malignant hematological tumors [11].

In this context, we used a large set of histological samples of CRC methylation datasets to screen for methylation sites that can effectively diagnose CRC and explored the relationship between the methylation degree, gene transcript, and protein expression. We identified three loci, cg02604524, cg07628404, and cg27364741, whose hypermethylation was associated with poor survival outcomes. In addition, we found that not all hypermethylated genes were associated with reduced gene expression, and that selected hypermethylation sites could effectively diagnose CRC. In the future, further detection and validation of these methylation sites in serum or stool samples will provide the means for non-invasive diagnosis of CRC.

## Materials and methods

### Data collection

We collected Illumina 450 K methylation array level and clinical data of CRC using TCGA (https://portal.gdc.cancer.gov/) database and downloaded the data using the Genomic Data Commons (GDC) data transfer tool recommended by TCGA website. The degree of CpG methylation was expressed using β values ranging from 0 to 1 (β = intensity of the methylated allele [M]/intensity of the unmethylated allele [U] + M + 100) .We used Python 3.7 (https://www.python.org/) to perform preliminary processing of the downloaded raw data. If the detected methylation site had a deletion value > 5%, the site was deleted. The “impute” package of R (https://bioconductor.org/packages/release/bioc/html/impute.html) was used to replace a small number of missing values by calculating the nearest neighbor average. The enabled function was “impute.knn.” We used the number of neighbors commonly used in the allocation, which is 10 (K = 10).

### Differential methylation sites and enrichment analysis

The “edgeR” package of R (https://bioconductor.org/packages/release/bioc/html/edgeR.html) was used to screen the differential methylation sites between the control and cancer groups. The criteria for differential methylation sites were defined as follows: (1) the foldchange of the cancer group was > 2.5 or < 0.4 that of the control group, with *P* < 0.05, and (2) the sites with an average β value < 0.1 were hypomethylated sites. At the hypermethylated sites, the mean β value should be > 0.2. The enrichment analysis was conducted for the genes in both the hypermethylated and hypomethylated sites. The GO (Gene Ontology) and KEGG (Kyoto Encyclopedia of Genes and GenomesGene Ontology) enrichment analysis was performed using the “clusterProfiler” of R (https://www.bioconductor.org/packages/release/bioc/html/clusterProfiler.html) software package. The enrichment pathway setting criteria were pvalueCutoff = 0.05 and qvalueCutoff = 0.2. The key core functions used were “enrichGO” and “enrichKEGG”.

### Screening of methylation sites and unsupervised clustering

We analyzed the significance of the differential methylation sites related to patient survival and screened out those having significant associations. Statistically significant loci met the screening criteria. Cases were clustered based on the methylation sites of survival significance. The latest version of “NbCluster” 3.0.1 (https://cran.r-project.org/web/packages/NbClust/index.html) provides 30 indicators to determine the the optimal number of clusters for 289 cases of CRC and the “ConsensusClusterPlus” package of R (https://www.bioconductor.org/packages/release/bioc/html/ConsensusClusterPlus.html) to cluster these 289 CRC cases into different clusters. The specific contents of the 30 indicators can be obtained from the instruction manual of “NbCluster”(http://mirrors.pku.edu.cn/CRAN/web/packages/NbClust/NbClust.pdf).

### Dimension reduction

We used the “FSelector” package (https://cran.r-project.org/web/packages/FSelector/index.html) of R to calculate the importance of methylation sites with survival significance to the classification of cancer and control groups and screened out the top eight important methylation sites. To avoid collinearity, we included eight methylation sites in ridge regression and found that three of these were statistically significant.

### Univariate diagnosis

For the three methylation sites obtained using dimensionality reduction, receiver operating characteristic curve (ROC) analysis was performed separately, and the area under the curve (AUC) was calculated. Data from 292 patients with CRC were obtained from TCGA. The GSE131013 (colon cancer) dataset from the Gene Expression Omnibus (GEO) database contained 144 control samples and 96 tumor samples.

### Machine learning model and classification

We screened hypermethylated loci from the TCGA database of CRC methylation data. Four machine learning models were established using the methylation sites mentioned above in the TCGA database and GSE131013 dataset. We constructed four classification models: logistic regression, NaiveBayes, MultilayerPerceptron, and RandomForest. We evaluated the accuracy of each model using 10-fold cross-validation, and the model with the highest accuracy had the best diagnostic performance. The machine learning models were established using Weka 3.8.5 software (https://www.filehorse.com/download-weka/58926/).

### Relationship between the degree of methylation and gene mutation

We investigated whether the degree of overall methylation affects gene mutations. Nine genes were included in this study. Three genes (*DPY19L2P1*, *EFCC1*, and *OTX1*) were found among the genes of the three methylation sites obtained through the above dimensionality reduction. The next three genes (*SLX4IP*, *GLRX*, and *SMAD3*) were determined among the genes where the three differential methylation sites with the lowest average methylation degree were located. The last three genes (*MDFI*, *C8orf34*, and *USP44*) were found among the genes where the three differential methylation sites with the highest average methylation degree were located. Non-coding genes were excluded from the analysis. The degree of methylation was divided into relatively high and relatively low methylation groups according to the median value.

### Correlation of methylation and estimation of immune cell infiltration

Immune cell infiltration in colorectal cancer patients was estimated from TCGA database using TIMER2.0 online analysis (http://timer.comp-genomics.org/). The top two immune cells with the largest correlation coefficients were selected, and the heat map of the correlation coefficients was drawn. The “ggcorrplot” package of R (https://cran.r-project.org/web/packages/ggcorrplot/index.html) visualizes the correlation coefficients.

### Statistical analysis

We confirmed the influence of the β value on survival using univariate Kaplan–Meier survival analysis. Cox multivariate analysis was used to identify the factors that affect survival, and the “survminer” package of R (https://cran.r-project.org/web/packages/survminer/index.html) was used for survival analysis. Pearson correlation was used for correlation analysis. Statistical significance was set at *P* < 0.05.

## Results

### Methylation sequencing data and clinical data

There were 485,577 methylation CpG sites in the 450 K methylation sequencing data, including 37 cases in the control group and 292 in the cancer group. After the deletion of the sites with a deletion value > 5%, 394,316 methylation CpG sites remained in the 450 K dataset for subsequent screening.

**Fig. 1:**
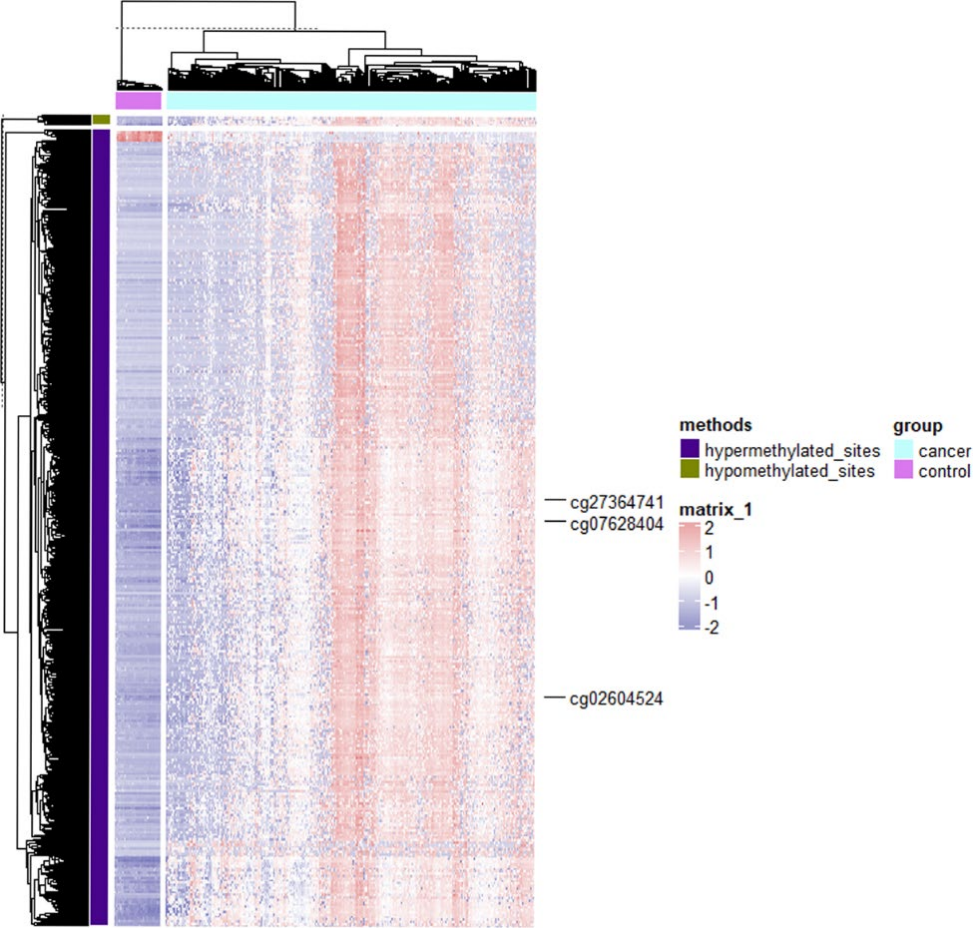
Heat map of the differential methylation degree values for different samples

In total, 4366 sites were screened (change > 2.5-fold [H group] or < 0.4-fold [L group]; *P* < 0.05) (Fig. 1 and Supplementary Table 5). The clinical information of the 292 patients included in the study is provided in Table 1. The number of men included in the study was slightly higher than that of women, and white was the largest ethnic group (68.5%). Adenocarcinoma was the most common type of cancer, and the tumor types were primarily stage II (39%) and stage III (27.4%). The cancer foci were primarily in the cecum (23.6%) and sigmoid colon (22.6%).

**Table 1:** Clinical characteristics of colorectal cancer patients

Clinical classification	450 K dataset	Percentage
**Age**		
≤ 65	135	46.2%
> 65	152	52.1%
Not reported	5	1.7%
**Gender**		
Female	133	45.5%
Male	154	52.7%
Not reported	5	1.7%
**Race**		
American Indian or Alaskan native	1	0.3%
Asian	11	3.8%
Black or African American	57	19.5%
White	200	68.5%
Not reported	23	7.9%
**Primary diagnosis**		
Papillary adenocarcinoma	1	0.3%
Mucinous adenocarcinoma	12	4.1%
Adenocarcinoma	107	36.6%
Not reported	172	58.9%
**Tumor stage**		
Stage I	45	15.4%
Stage II	114	39.0%
Stage III	80	27.4%
Stage IV	38	13.0%
Not reported	15	5.1%
**Site of resection or biopsy**		
Ascending colon	58	19.9%
Cecum	69	23.6%
Colon	47	16.1%
Descending colon	14	4.8%
Hepatic flexure of colon	13	4.5%
Sigmoid colon	66	22.6%
Splenic flexure of colon	5	1.7%
Transverse colon	14	4.8%
Not reported	6	2.1%

**Fig. 2:**
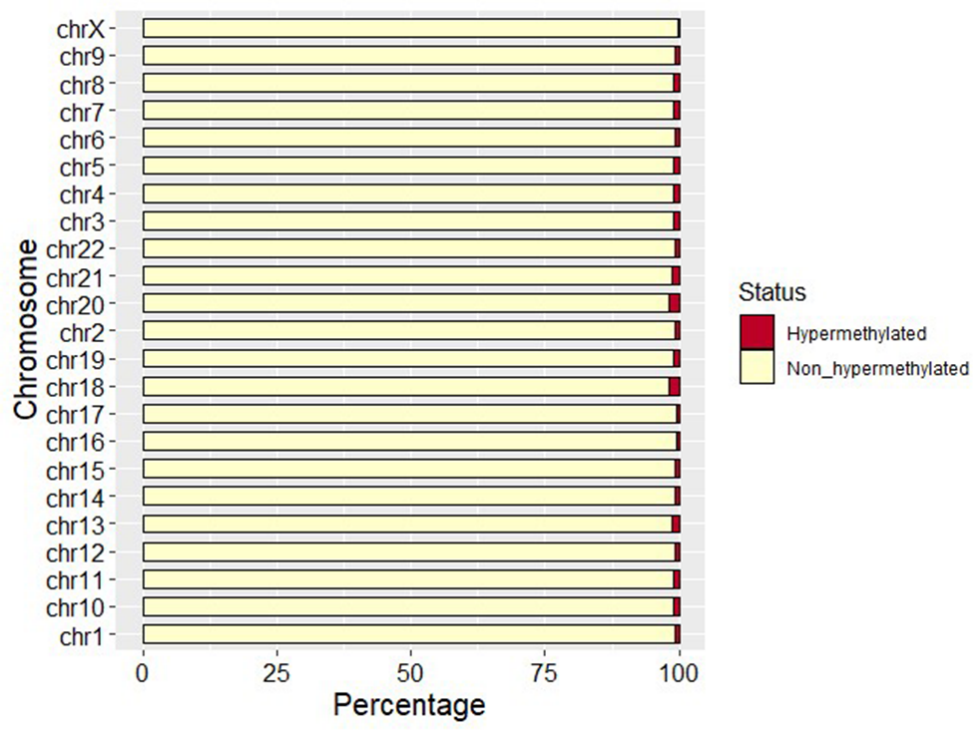
Percentage of hypermethylated sites on each chromosome

Chromosomes with the most hypermethylated sites were chr1, chr7, and chr2 (Supplementary Table 2). For autosomal chromosomes, the overall percentage of hypermethylated loci was 0.917% (Fig. 2). Chromosome 20 had the highest percentage of hypermethylation sites, reaching 1.760%, which was higher than the overall average (*P < 0.05*). The percentage of hypermethylation loci on chr17 was the lowest (0.495%), which was lower than the overall average (*P* < 0.05). For sex chromosomes, the X chromosome hypermethylation rate was very low (0.0449%).

### Enrichment analysis of genes with differential methylation sites

**Fig. 3:**
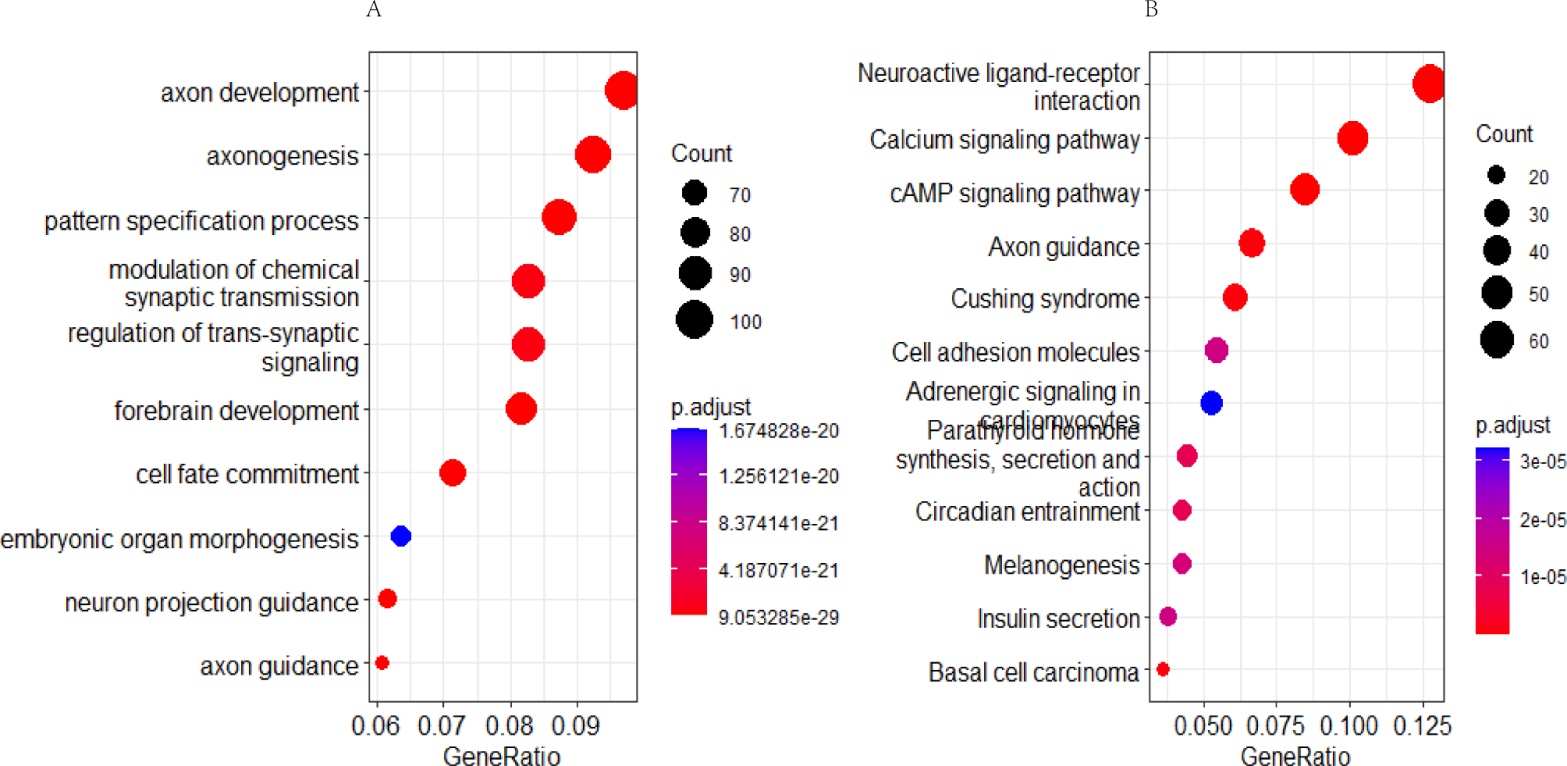
(**A**) GO enrichment analysis of hypermethylation site genes. (**B**) KEGG enrichment analysis of hypermethylation site genes

According to Gene Ontology (GO), the main enrichment pathways of the genes with hypermethylation sites were axon development, axonogenesis, and pattern specification processes (Fig. 3A). However, the Kyoto Encyclopedia of Genes and Genomes (KEGG) suggests that the main enrichment pathways are neuroactive ligand–receptor interaction, calcium signaling, and cAMP signaling (Fig. 3B). However, no enrichment pathway was found in the genes where the hypomethylation sites were located.

### Screening of potential diagnostic sites

**Fig. 4:**
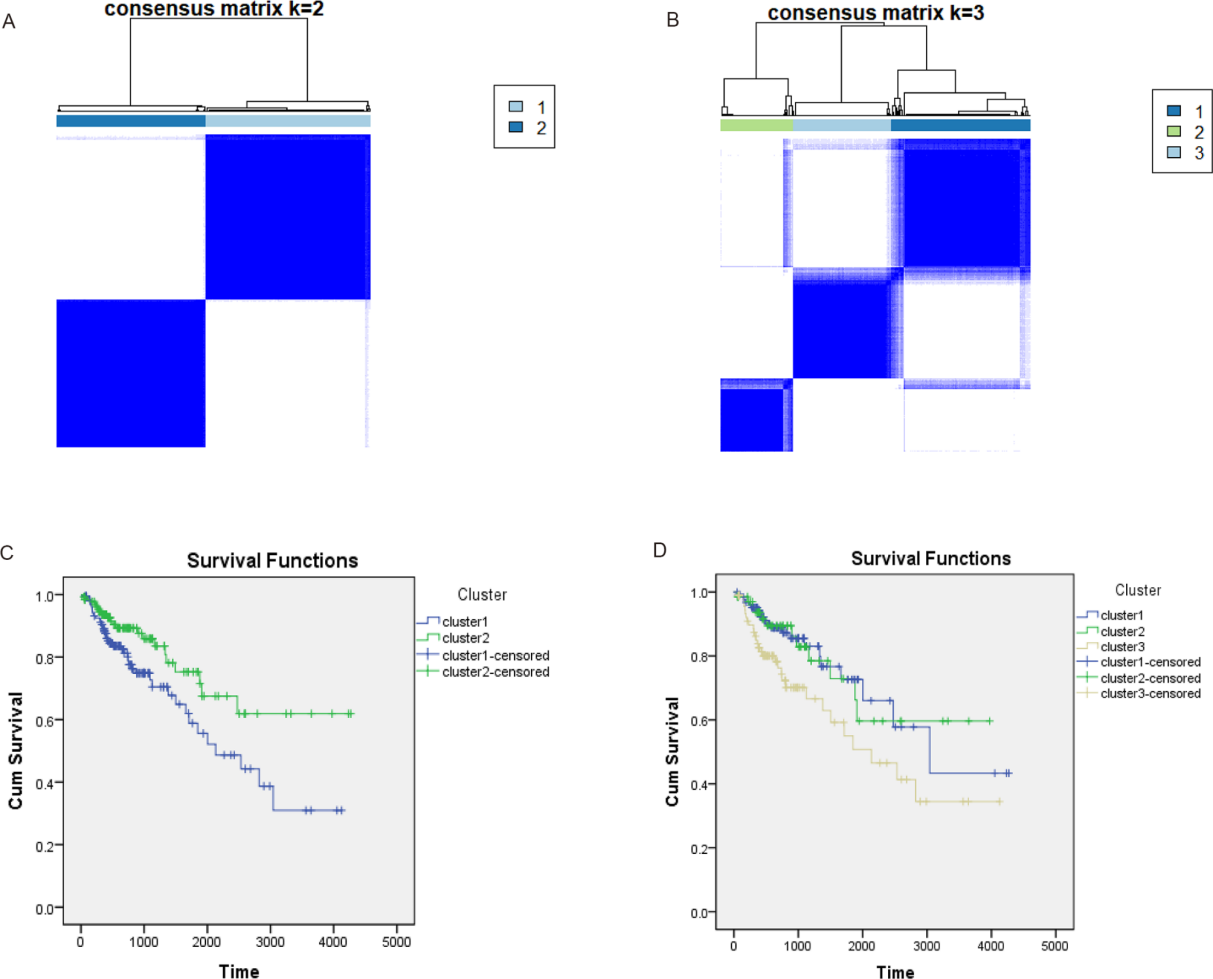
Cluster analysis results. (**A**) Cases were distributed into two groups via the k-means method; (**B**) Cases were distributed into three groups via the k-means method; (**C**) Survival analysis was performed between the two groups; (**D**) Survival analysis was performed for the three groups

Survival analysis revealed that there were survival differences between the high and low values of 91 loci (β values). Among the 30 indicators, twelve indicators recommended two clusters, and nine indicators recommended three clusters (Supplementary Fig. 1). When divided into two clusters using the k-means clustering method, cluster 1 had 155 samples and cluster 2 had 134 samples (Fig. 4A). Survival analysis revealed that cluster 2 was better than cluster 1 (Fig. 4C, P < 0.05, Supplementary Table 3). When divided into three clusters, cluster 1 had 128 samples, cluster 2 had 68 samples, and cluster 3 had 93 samples (Fig. 4B). Survival analysis revealed that clusters 1 and 2 were superior to cluster 3 (Fig. 4D, P < 0.05, Supplementary Table 4).

### Dimension reduction and phenotypic analysis

**Fig. 5:**
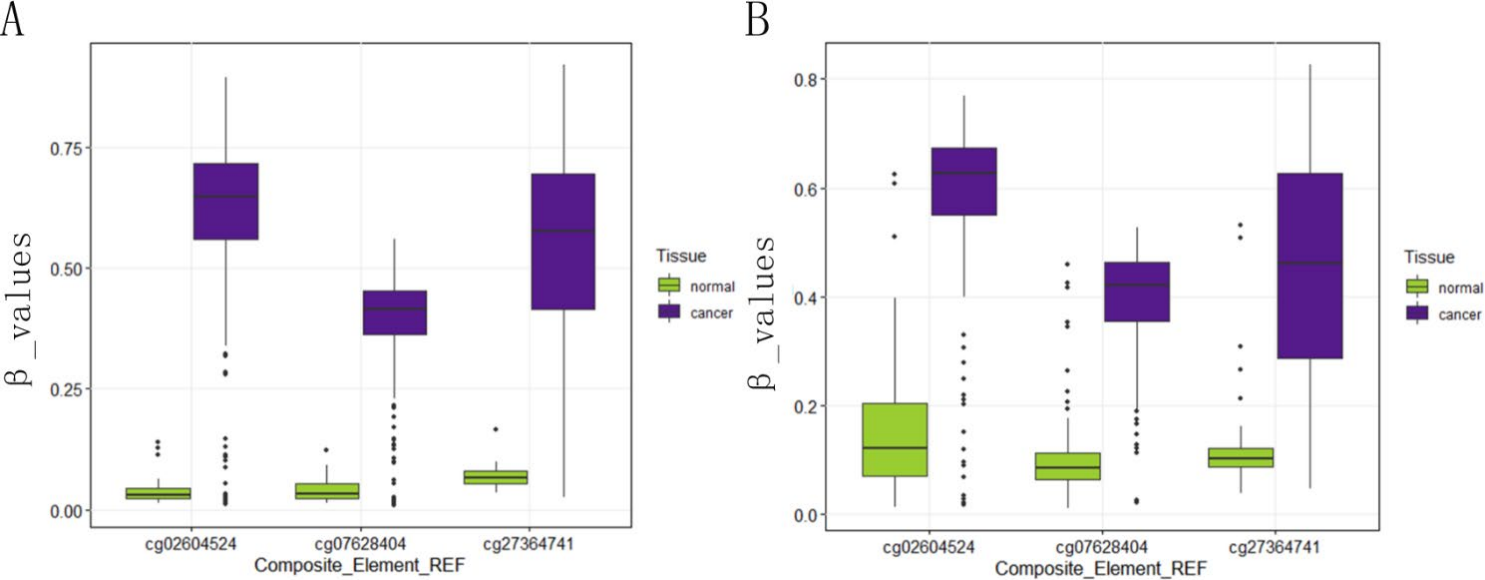
Methylation degree distribution at the three loci in the colorectal cancer group vs. control group in different datasets. (**A**) The Cancer Genome Atlas colorectal dataset; (**B**) The Gene Expression Omnibus GSE131013 dataset

Subsequently, we filtered out the first eight sites with the highest weight (Supplementary Table 5). We identified three meaningful loci (cg02604524, cg07628404, and cg27364741). The β values of the three potential methylation sites in TCGA colorectal dataset in the control and cancer groups are shown in Fig. 5A. The β values of the three loci in the GSE131013 dataset are shown in Fig. 5B (Supplementary Table 6). The β values of two datasets in the cancer group were significantly higher than those in the control group (*P* < 0.05).

### Univariate and multivariate survival analyses

**Fig. 6:**
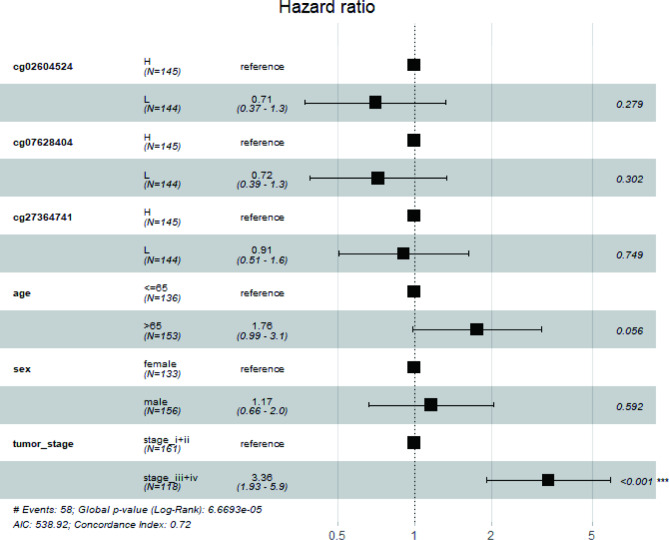
Multivariate survival analysis of colorectal cancer

Univariate analysis revealed that the survival time of the low-value group of the three loci (cg02604524, cg07628404, and cg27364741) was longer than that of the high-value group (*P* < 0.05, Supplementary Fig. 2). Cox risk regression analysis (Fig. 6) revealed that the survival time of stage I and stage II cancer patients was longer than that of stage III and stage IV cancer patients (*P* < 0.05, Supplementary Table 7).

### Single factor diagnosis

We used the ROC method to classify 292 CRC cases in the 450 K dataset. The AUC values of the three methylation sites cg02604524, cg07628404, and cg27364741 were 0.946, 0.970, and 0.947, respectively, in the TCGA dataset (Supplementary Fig. 3A), whereas in the GSE131013 dataset, they were 0.913, 0.957, and 0.908, respectively (Supplementary Fig. 3B).

### Machine learning diagnosis

Using the three methylation sites, cg02604524, cg07628404, and cg27364741, a machine learning model was established in the GSE131013 dataset. Ten-fold cross-validation showed that the accuracies of the classification of logical regression, NaiveBayes, MultilayerPerceptron, and RandomForest, were 93.8%, 95.0%, 94.6%, and 94.2%, respectively.

TCGA methylation data set of CRC was used for 10-fold cross-validation, and the accuracies were 98.8%, 99.4%, 98.8%, 99.4% for logical regression, NaiveBayes, MultilayerPerceptron, and RandomForest, respectively.

### Methylation and mutation

**Fig. 7:**
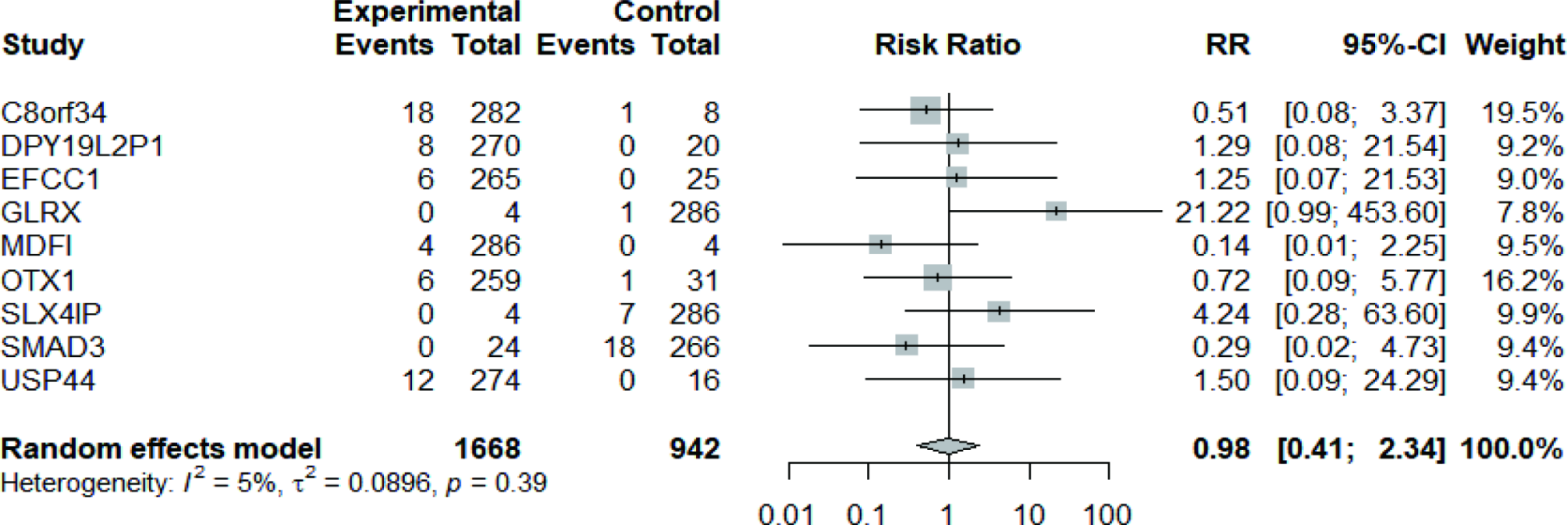
Mutation risk analysis of nine genes in the hypermethylated vs. hypomethylated groups

To investigate whether hypermethylation affects the frequency of gene mutations, we performed a risk analysis between the relative hypermethylated group and the relative hypomethylated group. The total number of gene mutations in the relative hypermethylation group of nine genes in 290 patients was 54. However, in the relative hypomethylation group, the total number of genetic mutations in the nine genes was 28. Overall, no significant difference was observed in the frequency of gene variation between the relative hypermethylation group and the relative hypomethylation group for the nine genes (relative risk: 0.98 [95% confidence interval: 0.41–2.34]; *p* > 0.05; Fig. 7).

### Methylation and gene expression

A weak significant correlation was observed between the degree of methylation of cg07628404 and *DPY19L2P1* (where cg07628404 is located) mRNA expression (Supplementary Fig. 4A; mRNA expression values and methylation CRC data were obtained from TCGA database). No significant correlation was observed between the methylation of cg02604524 and *EFCC1* expression where cg02604524 is located (Supplementary Fig. 4B). cg27364741 methylation was positively correlated with *OTX1* expression (*P* < 0.05) where cg27364741 is located (Supplementary Fig. 4C).

### Methylation and immune infiltration estimations

**Fig. 8:**
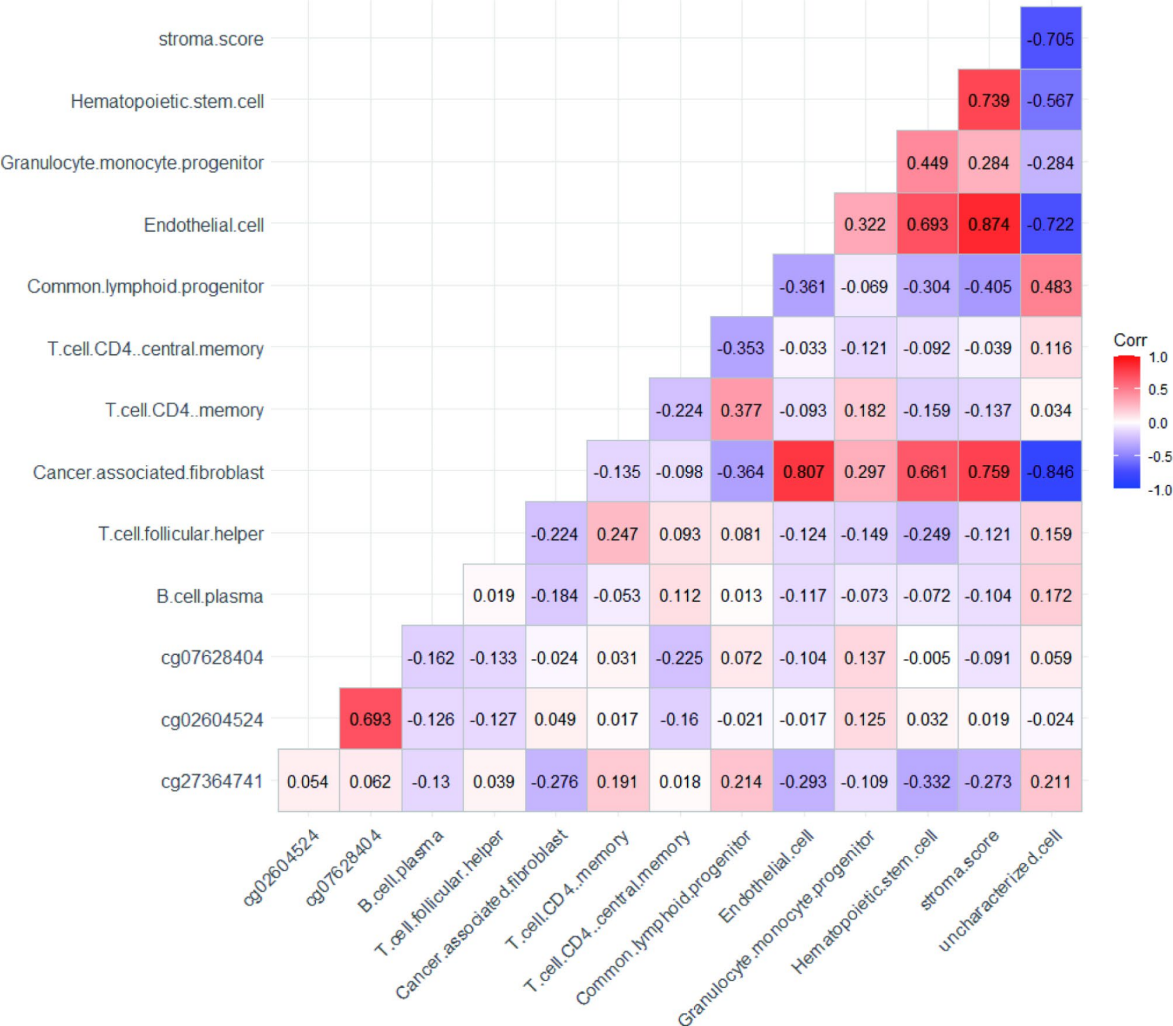
Heat map of correlation coefficients between three methylation sites and estimates of major immune cell infiltration

The three methylation sites were weakly correlated with different estimates of immune cell infiltration (Fig. 8). The correlation analysis revealed a weak negative correlation between cg02604524 and CD4 central memory T cell (r = -0.16, *P* < 0.05). Cg07628404 was weakly negatively correlated with CD4 central memory T cell (r = -0.22, *P* < 0.05) and B cell plasma (r = -0.16, *P* < 0.05). Cg27364741 was weakly negatively correlated with hematopoietic stem cells (r = -0.33, *p* < 0.05) and endothelial cells (r = -0.29, *P* < 0.05). Cg27364741 was positively correlated with common lymphoid progenitors (r = 0.21, *P* < 0.05) and uncharacterized cells (r = 0.21, *P* < 0.05).

## Discussion

Epigenetics is a gene expression regulation process that does not alter the DNA sequence [23]. Epigenetic processes include chromatin remodeling, histone changes, DNA methylation, and non-coding RNA expression [24]. DNA methylation changes, including significant hypermethylation, are more frequent in early colon tumors than previously thought [25]. Microorganisms in the gut can also affect DNA methylation, repair, and damage [26]. DNA methylation, histone modification, chromatin remodeling, nucleosome localization, non-coding RNA, and precise microRNA regulation are important epigenetic markers in progressive cancer subtypes [26]. Epigenetic modifications can also lead to tumor immune escape or impede immune monitoring, thereby playing an important role in tumor progression [27]. Finally, epigenetic changes can affect cell behavior and contribute to cancer development and progression [28].Our enrichment analysis revealed interesting things. Hypermethylation sites have multiple gene enrichment pathways, primarily related to the genesis and development of axons and nerves. However, genes with hypomethylation sites have no enrichment pathways. The pathway components in which the hypermethylation sites are located may have strong one carbon unit metabolism, and hypermethylation often inhibits gene expression. It is worth exploring whether there is a situation where the activity of the related pathways is inhibited.

Emerging non-invasive DNA methylation biomarkers are important for cancer prognosis and drug response [29]. Our cluster analysis revealed that subgroups with poor prognosis can be divided into groups of two or three, which may be helpful in judging clinical prognosis. Univariate survival analysis revealed that hypermethylation (cg02604524, cg07628404, and cg27364741) indicated a poor survival prognosis.

In this study, we screened as few methylation sites as possible from a large number of methylation sites that can distinguish between cancer and normal groups. In contrast to that of other studies [30], we used the random forest method to calculate the ability of different sites to distinguish between the cancer and control groups. To avoid the influence of collinearity, we adopted the ridge regression method and finally screened three sites. The method for screening methylation sites in this study was simple and efficient. Using only the three above-mentioned sites, the cancer and control groups could be well distinguished by establishing a NaiveBayes model.

Many studies have been conducted to establish a classifier based on the gene signatures of tumors. A Logit model was developed for the diagnosis of colon cancer using specific volatile organic compounds in stool, with a sensitivity of 87.9% and specificity of 84.6%. Using logical regression modeling with five serum peptide markers, the diagnosis of colon cancer showed a sensitivity of 82% and specificity of 93% [31]. A calibrated logical regression classifier was used to classify central nervous system tumors, and the results showed 76% (838/1104) consistency between DNA methylation classification and histopathological classification. Using the classifier based on DNA methylation, 15.5% (171/1104) of the cases was classified into an unambiguous molecular subgroup, which was not possible based on histopathology [31]. A panel of 13 methylated markers could also be effective in the diagnosis of colon cancer; however, the selection of methylated markers in this study was excessive, and the dimensionality reduction process was complicated [30]. The established NaiveBayes model with high accuracy and few characteristic loci has achieved good results in both independent samples and 10-fold cross-validation.

The degree of methylation in the nine genes included in this study was not a risk factor for mutations. However, mutations in driver genes are closely related to the changes in DNA methylation [32]. In CRC, the BRAF-V600E mutation that recruits DNA methyltransferase 3 beta to a target on the CpG island promoter leads to DNA hypermethylation. According to the analysis of the nine genes and relevant reports, methylation and gene mutation were not common phenomena; they might be special individual phenomena.

Changes in the epigenome drive an abnormal transcription program, which promotes the occurrence and development of cancer [33]. Epigenetic changes, such as promoter hypermethylation, may lead to cancer through the inactivation of tumor suppressor genes [34]. While epigenetic changes are associated with the occurrence and development of cancer, tumor-related epigenomic changes are not the main carcinogenic factors [35]. Hypermethylation of *GABRA2*, *ZNF257*, and *SLC5A8* is associated with their decreased expression [36]. In our study, a weak correlation was observed between the selected methylation sites and the relevant immune cell infiltration. This suggests a link between methylation levels and immune cell infiltration. In colorectal cancer, DNA methyltransferase DNMT3A was found to be associated with infiltration of six major immune cells [37]. One study revealed that NEFM DNA methylation was moderately to strongly negatively associated with infiltration levels of B cells, CD8 + T cells, CD4 + T cells, macrophages, neutrophils, and dendritic cells [34]. This suggests that there may be a relationship between gene methylation and the infiltration of immune cells. The degree of methylation can be used for clinical classification of bladder cancer, and each subtype has different immune scores and survival differences [38]. In our methylation sequencing, we found that the degree of methylation in most samples of the cancer group was higher than that in normal controls. Persistent epigenetic changes caused by hypermethylation or hypomethylation can be used as effective biomarkers for cancer diagnosis and treatment [26]. Thus, the prospects for drugs that target epigenetic factors are very promising [28].

### Limitations

In future studies, a large number of CRC samples should be collected to verify the diagnostic performance of the above-mentioned methylation loci. Patient compliance and the number of case specimens collected also limited the progress of this diagnostic test. By optimizing the screening of methylation markers, more of these may be identified as candidates for the diagnosis and treatment of CRC in the future.

Owing to the lack of precancerous lesion data in TCGA database, we were temporarily unable to perform the classification of adenoma and CRC; the establishment of a machine learning model to effectively distinguish precancerous lesions from CRC is of great interest. Moreover, independent methylation validation datasets for rectal cancer are lacking in this paper, and it is expected that appropriate datasets will be used for further validation.

## Conclusions

Hypermethylation sites were distributed at different frequencies in chromosomes. The hypermethylated genes are primarily enriched in pathways involved in axon and neural development. In the biopsy tissue samples, selected hypermethylated sites could effectively diagnose CRC, and the accuracy of the NaiveBayes model in CRC diagnosis was outstanding. Hypermethylation of cg02604524, cg07628404, and cg27364741 and advanced tumor stage were associated with poor survival outcomes; however, not all hypermethylated genes were associated with reduced gene expression. Hypermethylation of genes may be an incidental event in genetic variation. Three methylation sites were weakly correlated with individual immune cell infiltration. Hypermethylation sites may be a treasure chest for the development of future diagnostic markers for colorectal cancer.

## Electronic supplementary material

Below is the link to the electronic supplementary material.

Supplementary Material 1

Supplementary Material 2

Supplementary Material 3

Supplementary Material 4

Supplementary Material 5

Supplementary Material 6

Supplementary Material 7

Supplementary Material 8

Supplementary Material 9

Supplementary Material 10

Supplementary Material 11

## Data Availability

All the data were downloaded from TCGA (https://portal.gdc.cancer.gov/) and GEO websites (https://www.ncbi.nlm.nih.gov/geo/query/acc.cgi?acc=GSE131013), as well as from the Supplementary Materials.
