# Supplementary figures and images for: Optimized screening of DNA methylation sites combined with gene expression analysis to identify diagnostic markers of colorectal cancer
Source: BMC Cancer. 2023 Jul 3;23:617. doi: 10.1186/s12885-023-10922-2 (PMC10318760; doi:10.1186/s12885-023-10922-2)

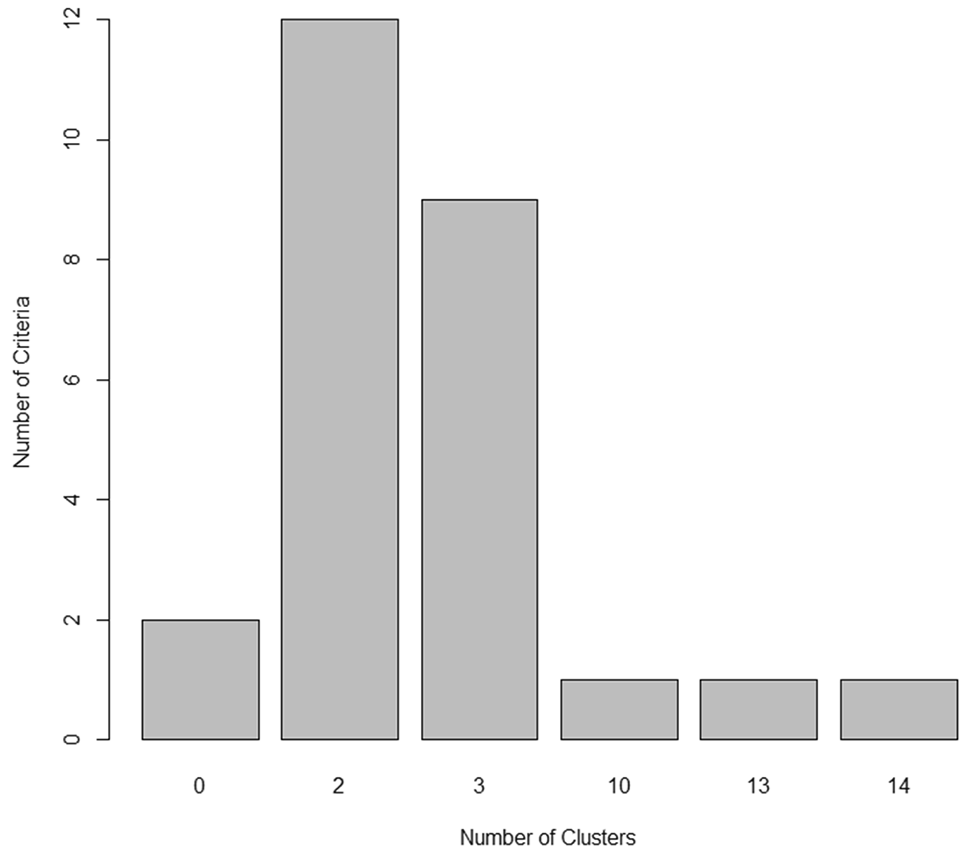

Supplement: Supplementary file 1 — Supplementary Material 1 [file 12885_2023_10922_MOESM1_ESM.png]

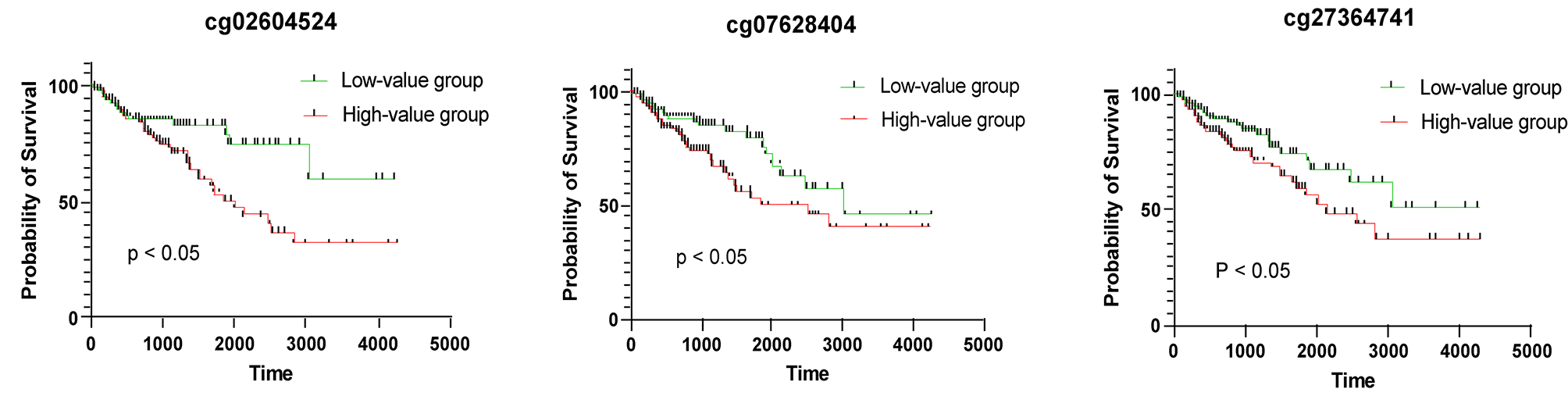

Supplement: Supplementary file 2 — Supplementary Material 2 [file 12885_2023_10922_MOESM2_ESM.png]

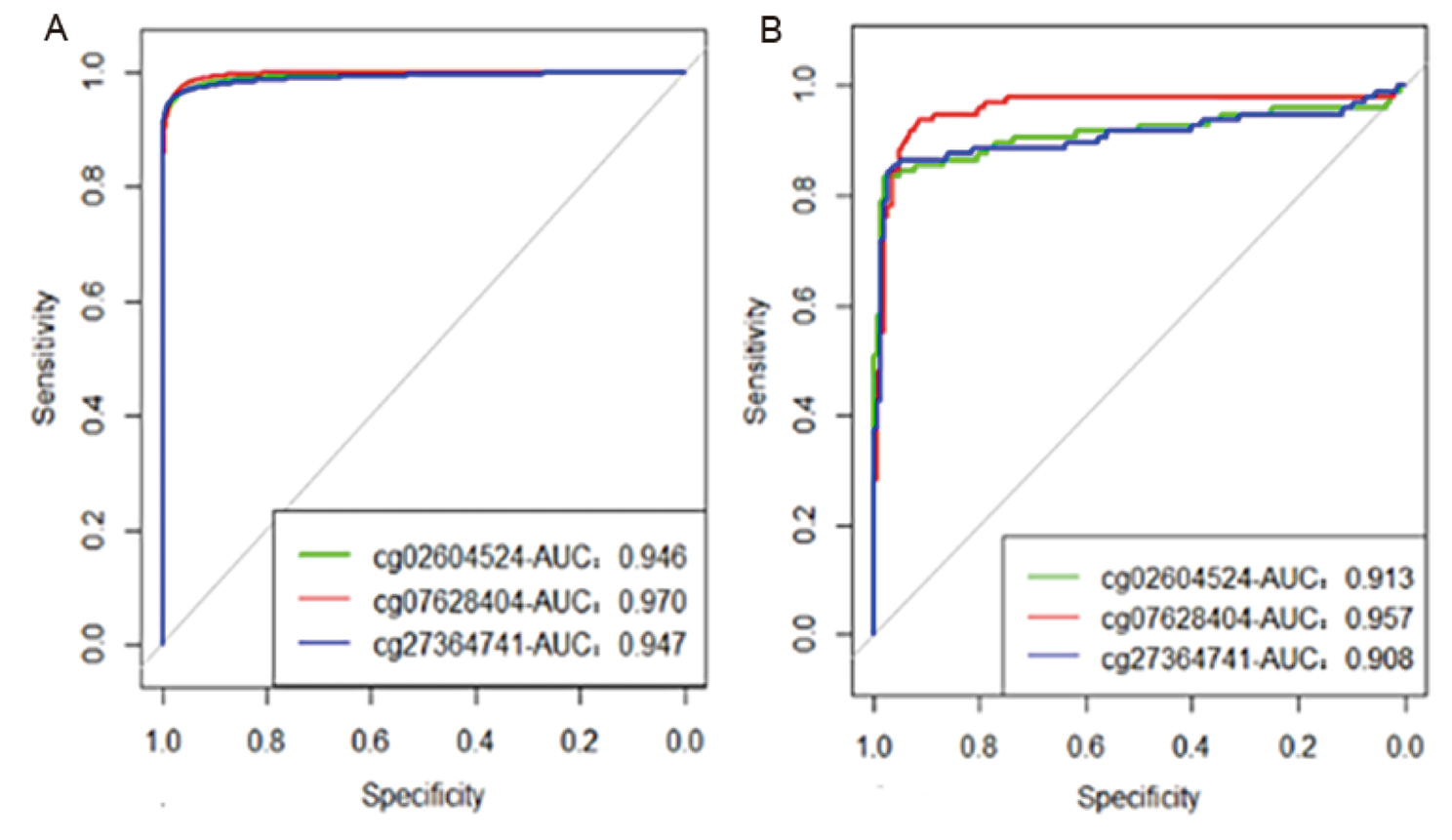

Supplement: Supplementary file 3 — Supplementary Material 3 [file 12885_2023_10922_MOESM3_ESM.png]

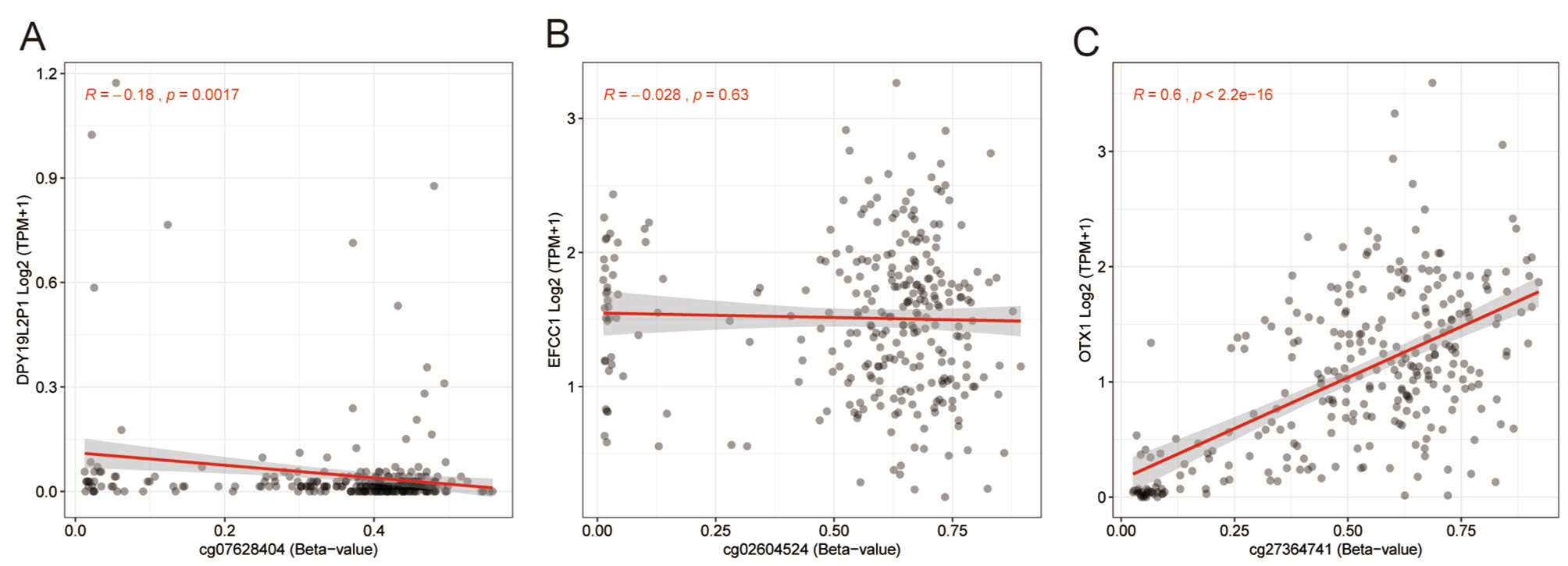

Supplement: Supplementary file 4 — Supplementary Material 4 [file 12885_2023_10922_MOESM4_ESM.png]
